# Biocatalysis in the Chemistry of Lupane Triterpenoids

**DOI:** 10.3390/molecules26082271

**Published:** 2021-04-14

**Authors:** Jan Bachořík, Milan Urban

**Affiliations:** 1 Department of Organic Chemistry, Faculty of Science, Palacký University in Olomouc, 17. listopadu 12, 771 46 Olomouc, Czech Republic; jan.bachorik01@upol.cz; 2 Medicinal Chemistry, Faculty of Medicine and Dentistry, Institute of Molecular and Translational Medicine, Palacký University in Olomouc, Hněvotínská 5, 779 00 Olomouc, Czech Republic

**Keywords:** lupane, betulinic acid, betulin, lupeol, biocatalysis, extraction, biotransformation, synthesis, prodrugs, enzyme

## Abstract

Pentacyclic triterpenes are important representatives of natural products that exhibit a wide variety of biological activities. These activities suggest that these compounds may represent potential medicines for the treatment of cancer and viral, bacterial, or protozoal infections. Naturally occurring triterpenes usually have several drawbacks, such as limited activity and insufficient solubility and bioavailability; therefore, they need to be modified to obtain compounds suitable for drug development. Modifications can be achieved either by methods of standard organic synthesis or with the use of biocatalysts, such as enzymes or enzyme systems within living organisms. In most cases, these modifications result in the preparation of esters, amides, saponins, or sugar conjugates. Notably, while standard organic synthesis has been heavily used and developed, the use of the latter methodology has been rather limited, but it appears that biocatalysis has recently sparked considerably wider interest within the scientific community. Among triterpenes, derivatives of lupane play important roles. This review therefore summarizes the natural occurrence and sources of lupane triterpenoids, their biosynthesis, and semisynthetic methods that may be used for the production of betulinic acid from abundant and inexpensive betulin. Most importantly, this article compares chemical transformations of lupane triterpenoids with analogous reactions performed by biocatalysts and highlights a large space for the future development of biocatalysis in this field. The results of this study may serve as a summary of the current state of research and demonstrate the potential of the method in future applications.

## 1. Introduction

Natural products have been used in traditional medicine for a long time, and since the appearance of modern science, they have been recognized as valuable sources of new drugs [1,2,3,4,5]. Considerable money has been invested in their isolation from natural sources and characterization of their structures and biological activities. High-throughput screening was introduced for fast and efficient testing of large numbers of new compounds from nature or chemistry labs [6,7,8,9]. In 2015, David et al. wrote a very informative review in which the authors summarized some of the most successful drug leads in history that came from natural products [10]. This review also reports that there was a significant decline in investment in natural drug discovery programs during the early 2000s because more attention was paid to combinatorial chemistry and rational drug design, which appeared to be considerably more promising. Pharmaceutical companies expected new methods to enable the production of a number of new drugs, aiming at new molecular targets that may be game changers for many diseases. The new approach, however, has exhibited lower productivity in bringing new drugs to the market than previously expected, and over the years, some of its disadvantages have appeared. One of these drawbacks is that rational design and solid-phase combinatorial chemistry usually use limited structural variability, which also limits the outcome [11]. Natural compounds, on the other hand, have almost unlimited variability of molecules, and they occupy a considerably larger chemical space than the more focused libraries obtained as a result of the previously mentioned methods [11]. Although it is often difficult to obtain a unique active molecule from natural material and uncover its structure and activity, in many cases, this effort has paid off, leading to a commercial drug. Within the past several years, the search for new natural products started to reemerge as a source of new drugs [10]. In our opinion, equilibrium has been achieved between the two approaches, which are both important in the drug discovery process.

One of the largest and most important groups of natural products, which has attracted considerable attention from researchers, is terpenes. Hundreds of terpenes are isolated every year from natural resources, and even more are prepared by semisynthetic methods [12,13]. Terpenes can have a variety of different roles in living organisms; for example, they can participate in such processes as transferring messages and defending organisms [14,15,16].

**Figure 1:**
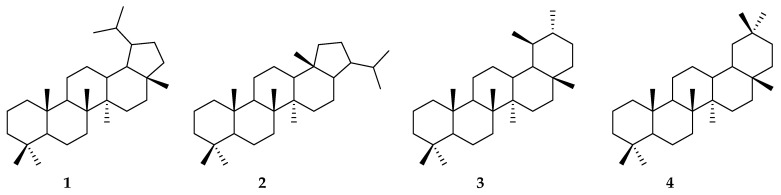
Examples of structural families of triterpenes—lupane (**1**), hopane (**2**), ursane (**3**), and oleanane (**4**).

Terpenes can be formally divided into smaller subclasses based on the number of carbons. Triterpenes are the subclass that contains 30 carbons in its structures. This subclass is composed of a large number of compounds that may be divided according to their basic skeletons into several structural families. The most important families of triterpenes are derivatives of protostane, cycloartane, dammarane, and euphane, and pentacyclic derivatives, such as oleanane, ursane, gammacerane, lupane, and hopane. Figure 1 shows the structures of selected main skeletons of pentacyclic triterpenes—lupane (**1**), hopane (**2**), ursane (**3**), and oleanane (**4**) [17,18].

**Figure 2:**
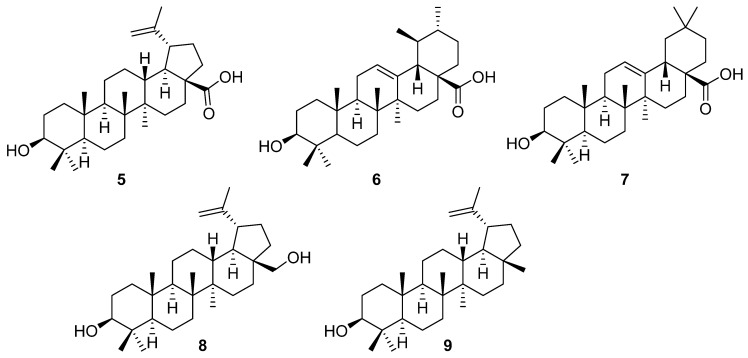
Chemical structures of betulinic acid (**5**), ursolic acid (**6**), oleanolic acid (**7**), betulin (**8**), and lupeol (**9**).

Triterpenes often have a variety of biological activities, and among them, betulinic acid (**5**) plays an important role, along with ursolic (**6**) and oleanolic acid (**7**) (Figure 2) [19,20,21,22,23,24,25,26,27].

The biological activities of betulinic acid (**5**) will be discussed later. However, the potential use of **5** as a commercially available drug may be limited by its insufficient solubility in water and low bioavailability [28,29]. This low bioavailability is also the main complication encountered when performing biological experiments; therefore, the optimization of pharmacological parameters, including solubility, is always an important part of the development of derivatives of acid **5**. Many studies have focused on structural modifications of betulinic acid (**5**) that improve its solubility and bioavailability, especially the enhancement of solubility in polar media, and many studies have also focused on the improvement of selectivity [29,30,31].

In this review, we mostly focus on the biosynthesis of lupane triterpenoids, including betulinic acid (**5**), biocatalyzed modifications of acid **5,** and its derivatives, and we compare those methods to classical approaches of synthetic organic chemistry that are commonly used. Our research group has been focused on the chemical modification of **5** for many years, and currently, we see biocatalysis as an important alternative for the preparation of new molecules for our biological studies, providing an alternative that affords chemical modifications that may not easily be obtained by classical synthetic approaches.

## 2. Discovery of Betulinic Acid (5) and Its Biological Activity

Betulinic acid (**5**) was first discovered in the methanolic extract of the plant *Gratiola officinalis* by Retzlaff in 1902 [32]. Notably, the biological activities associated with acid **5** were first reported much later, to the best of our knowledge, as first mentioned in 1976, when a chloroform extract of *Vauquelinia corymbosa* (containing betulinic acid) showed a growth-inhibitory effect against lymphocytic leukemia P-388 cells [33]. In 1994, Fujioka et al. [34] extracted betulinic acid (**5**) together with platanic acid from leaves of *Syzigium claviflorum*. Both compounds were identified as inhibitors of HIV replication in H9 lymphocyte cells. In 1995, betulinic acid (**5**) was found to be a selective inhibitor of human melanoma by Pisha et al. [35]. The growth of tumors was completely inhibited by the induction of apoptosis, and no toxicity was observed. Two years after this discovery, it was reported by Schmid et al. [36] that acid **5** also induced apoptosis in human neuroblastoma cell lines. In 2002, Freire et al. [37] studied dichloromethane extracts of the inner and outer barks of *Eucalyptus globulus*. Both barks showed different compositions according to their results. Betulinic acid (**5**) was the major component of the outer bark of *Eucalyptus globulus*. In the same year, betulinic acid (**5**) was also identified in *Rosmarinus officinalis* L. by Abe et al. [38]. The authors extracted compound **5** from leaves using MeOH. Last but not least, fungi are also an important source of lupane triterpenoids. Many fungal species have been used in traditional medicine for hundreds of years; among them, *Inonotus obliquus* plays an important role because of its significant anti-cancer activity, which is associated with the presence of triterpenoids including betulinic acid (**5**) and betulin (**8**) [39]. More information about its natural occurrence and biological activities may be found in the introductory part of a very recent review [40], which is mostly focused on derivatives of betulinic acid (**5**) with antiprotozoal activity.

## 3. Natural Sources of Betulinic Acid (5), Betulin (8), and Lupeol (9)

Betulinic acid (**5**) may be found in a number of plant species [40]; however, most of these plants contain the desired terpene **5** in amounts substantially less than 1%. One of the most common sources of **5** is the white part of birch bark (e.g., *Betula pendula*, *Betula alba*, *Betula platyphylla,* and *Betula pubescens*) [41,42]. In 2006, Zhao et al. [42] developed a method for the simultaneous extraction and determination of betulin (**8**) and betulinic acid (**5**) from white birch bark. Different solvents were used for the extraction of **5** and **8**, including dichloromethane, ethyl acetate, acetone, chloroform, methanol, and 95% ethanol. The best solvent for the extraction was ethanol. Determination was performed using RP-HPLC with a C_18_ column and a mobile phase of acetonitrile–water 86:14 (*v*/*v*). A UV detector (at λ = 210 nm) was used for detection. The results showed that the percentages of betulinic acid (**5**) and betulin (**8**) in white birch bark differed with the location of the tree growth site in China, and showed that the amount of betulinic acid (**5**) was usually lower than the amount of betulin (**8**). In a study from 2011, Ren and Omori described a simple method of extracting **8** in high purity from sycamore outer bark (*Platanus occidentalis*). First, the bark was peeled off by hand, and the bark was subsequently collected and crushed. Next, the crushed bark was washed with boiling water for 1.5 h. After filtration, terpenes were extracted with organic solvents three times (e.g., methanol, acetone, ethanol, and 2-propanol). Combined filtrates were evaporated, and the product was collected. The yields were between 5–6% (*w*/*w*). The amount of collected betulinic acid (**5**) depended mainly on the organic solvent used during the extraction phase. The best results were obtained by extraction with methanol (yield 5.70%). The purity of **5** was 95% [43]. In our lab, we have been obtaining acid **5** by the extraction of sycamore (*Platanus hispanica*) bark for several decades. We usually collect bark that spontaneously peels off of the trees during the summer and extract it directly with methanol. After 2–3 crystallization procedures, we usually obtain 1–2% (weight of the dry bark) betulinic acid (**5**) of 98% purity [44].

Mullally et al. [45] described a more sophisticated supercritical carbon dioxide extraction of **5** from *Souroubea sympetala* Gilg. This new method was compared with other extraction techniques, such as extraction with ethyl acetate, accelerated solvent extraction, ultrasonic-assisted extraction, and Soxhlet extraction. The concentration of **5** after supercritical carbon dioxide extraction was 5.54 ± 0.2 mg/g extract. This value was comparable to ethyl acetate extraction. The concentration of betulinic acid (**5**) was 6.78 ± 0.2 mg/g, which was the highest value. In 2013, Patinha and coworkers [46] studied the compositions of extracts of the inner and outer barks of *Eucalyptus grandis x globulus* by GC/MS. The results showed distinct compositions of the inner and outer bark. The outer bark was primarily composed of triterpenoids. The content of betulinic acid (**5**) was 626.0 mg kg^−1^ in the outer bark. Extraction with supercritical carbon dioxide was also performed for comparison. The results of this extraction showed that acetylated triterpenoid acids were more significantly extracted than free triterpenoid acids. In 2015, Liu et al. [41] described a new greener method for the extraction of betulinic acid (**5**) from birch bark using subcritical water as the extraction medium. The subcritical state of water can be reached under pressure at temperatures between 100 °C and 374 °C. Under these conditions, the thermal motion of water is extreme according to the authors of the article. This difference in motion leads to a change in the parameters of water. The dielectric constant of subcritical water mimics those of methanol and acetone at ambient temperature. Optimization of subcritical extraction was performed using response surface methodology, and under optimal conditions, the yield of betulinic acid (**5**) was 28.03 mg/10 g birch bark. The results showed that subcritical water extraction of **5** is an environmentally friendly, rapid, and selective method.

Hydrophobic deep eutectic solvents were used as an alternative for the extraction of betulinic acid (**5**) and other terpenic acids in work published in 2020 by Silva et al. [47]. Deep eutectic solvents are described in the article as a combination of at least one hydrogen bond acceptor and a hydrogen bond donor that forms a eutectic mixture. Extraction of terpenic acids from the outer bark of *Eucalyptus globulus* was accomplished using a combination of menthol and thymol (1:2) at room temperature. The extraction yields of terpenoid acids were 1.8 wt% for ursolic acid (**6**), 0.84 wt% for oleanolic acid (**7**), and 0.30 wt% for betulinic acid (**5**). Betulin (**8**), as mentioned earlier, is highly abundant in birch bark, and since its content is up to 30% [48], this source is dominant in industrial-scale extractions. There are many other plant species that produce betulin (**8**) in low amounts, but to the best of our knowledge, none of them are used as important sources of it. Lupeol (**9**) is another lupane triterpene naturally occurring in plants; however, its quantities are usually lower than the quantities of **5** or **8,** and it is usually obtained as a side-product of extractions of other triterpenes [49,50,51]. A basic summary of the main lupane triterpenoid sources is in Table 1.

## 4. Semi-Synthetic Procedures for the Preparation of Betulinic Acid (5)

**Scheme 1:**
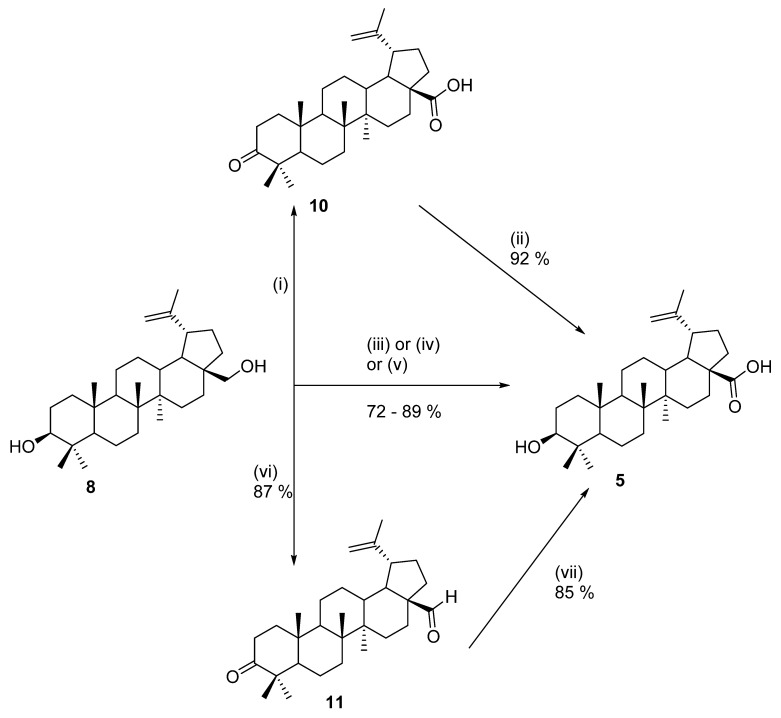
Methods of synthesis of betulinic acid [47,48,49,50,51,52]. Reagents and conditions: (i) chromium(VI) oxide; sulfuric acid, and acetone, H_2_O; (ii) sodium tetrahydroborate and isopropyl alcohol; (iii) 4-acetylamino-2,2,6,6-tetramethylpiperidine-N-oxyl, sodium chlorite, tetrabutylammomium bromide, sodium hypochlorite, and phosphate buffer at 50 °C; (iv) 4-acetylamino-2,2,6,6-tetramethylpiperidine-N-oxyl, tetrabutylammomium bromide, sodium hypochlorite, and phosphate buffer (pH = 7.6) at 50 °C; (v) BAIB, TEMPO, NaH_2_PO_4_, NaClO_2_, 2-methyl-2-butene, BuOAc, water, and t-BuOH at 20 °C for 6 h; (vi) 2,6,6-tetramethyl piperidine-N-oxyl, tetrabutylammomium bromide, and sodium hypochlorite in phosphate buffer with dichloromethane for 6 h with pH = 6.8; (vii) sodium dihydrogenphosphate, sodium permanganate, DCM, water, and tert-butyl alcohol at 25 °C for 3 h; (viii) K_2_CO_3_ and MeOH for 24 h; and (ix) KOH and MeOH with heating for 3 h.

Since betulin (**8**) is far more abundant in birch bark than betulinic acid (**5**) [42], and the only difference between these two molecules is the oxidation stage of carbon C-28, it is desirable to synthesize betulinic acid (**5**) by simple oxidation of the 28-CH_2_-OH functional group to 28-COOH. Making betulin (**8**) a common starting material for the chemical synthesis of acid **5** could decrease the price of the latter significantly. The main crux of the simple oxidation step is the presence of the 3*β*-hydroxy group that needs to be preserved. The chemical synthesis of betulinic acid (**5**) was described, for example, by Baltina et al. [52]. Betulin (**8**) was first extracted from the birch bark of *Betula pendula* with an extraction system of 2-propanol–water (9:1, *v*/*v*). Betulinic acid (**5**) was then prepared by a two-step procedure. The first step was Jones’ oxidation, which was followed by reduction with sodium borohydride in 2-propanol (Scheme 1). The yield of betulinic acid (**5**) was 92% after the crude product was recrystallized from hot methanol.

In 2006, Csuk and coworkers developed another synthetic route for the preparation of **5** from **8**. Betulin (**8**) was isolated from the bark of white birch (*Betula alba*). The bark contains up to 25% of **8**. Betulinic acid (**5**) was then prepared by TEMPO-mediated oxidation of **8** (Scheme 1). The yield of betulinic acid (**5**) was 86% [53]. Barthel et al. [54] used the same oxidation system for the oxidation of **8**, utilizing 4-acetamido-TEMPO. The exact reaction conditions are described in Scheme 1. The yield of betulinic acid (**5**) was 72% after recrystallization from ethanol.

Ressmann et al. [55] developed a new method for the extraction of betulin (**8**) and streamlined oxidation of extracted **8** to betulinic acid (**5**). The extraction method employed by these researchers is based on a biphasic system consisting of aqueous phosphonium hydroxide solution and n-butyl acetate. Using this system of solvents, betulin (**8**) could be extracted in high yields at room temperature after 1 h. Another advantage of this method is that after phase separation, the crude extract could be directly oxidized using TEMPO and hypervalent iodine (III) reagents (Scheme 1). The yield of betulinic acid was 18 wt.%, and after column chromatography, it was 22 wt.% after recrystallization from methanol/water.

Another method for the preparation of betulinic acid (**5**) was based on the oxidation of betulinal **11**. Betulinal **11** can be oxidized by NaMnO_4_ (Scheme 1). The yield of **5** after purification was 85%. Another oxidation method described by the authors for the oxidation of betulinal **11** was based on a combination of MnSO_4_/AgNO_3_. The yield of betulinic acid (**5**) after purification was also 85%. The third method of oxidation of betulinal **11** was performed by oxidation with MnO_2_. This method of oxidation produced betulinc acid (**5**) in a small yield of 18% [54]. In some cases, betulin (**8**) was firstly acetylated before the oxidation, and the free acid was then released by the reaction betulic acid acetate with K_2_CO_3_ with high yield [56,57]. Some alternative procedures for oxidation are included in [40].

Both betulin (**8**) and lupeol (**9**) are usually not prepared by semi-synthetic methods; they mostly come from extraction of natural materials.

## 5. Chemical Modification of Betulinic Acid (5)

The importance of chemical modification of betulinic acid (**5**) is highlighted in the introduction section of this article. This topic has been reviewed several times [40,58,59]. In addition, some specific reaction conditions will be mentioned in this chapter to compare them with biosynthetic methods that will be described later. In 2015, Kvasnica et al. published a review article covering methods of skeletal modification of pentacyclic triterpenes, including betulinic acid (**5**), with nitrogen and sulfur heterocycles. Authors covered the synthesis and biological activities of three-membered, five-membered, six-membered, and seven-membered terpenic heterocycles [58]. Another review covering advances in the modification of betulinic acid (**5**) was published in 2018 by Borkova et al. This article was focused on chemical modifications of ring A of **5**. The authors also covered the drug potential of the prepared derivatives of betulinic acid [59]. A more recent review paper focusing on the functionalization of betulinic acid (**5**) and its analogs was published two years ago by Sousa et al. [60]. Their review covers chemical modifications of triterpenic compounds by amination, hydroxylation, esterification, alkylation, sulfonation, alkyne-azide cycloaddition, and the palladium-catalyzed cross-coupling reaction and condensation reactions in different positions, and provides the reader information about the biological activities of the prepared derivatives. The authors also reviewed the synthesis of heterocyclic derivatives and polymer conjugates [60].

Much effort has been invested into the chemical transformation of carboxylic groups of betulinic acid (**5**) to produce amides. The preparation of amides is among the synthetic methods that in some cases may be easily replaced by enzymatic synthetic procedures. Amides are very important derivatives of betulinic acid and its close analogs, especially because of their high anti-HIV activity [61,62], anticancer activity [63,64], hepatoprotective effect [65], and notable self-assembly properties [66].

**Scheme 2:**
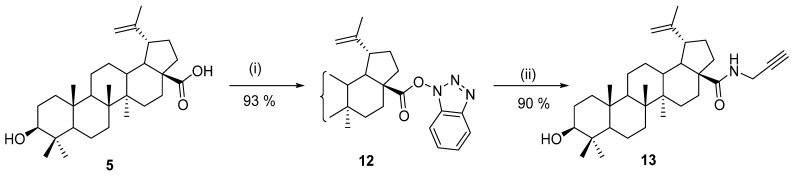
Preparation of a propargylamide derivative of betulinic acid [67]. Reagents and conditions: (i) TBTU, DIPEA, THF; (ii) propargylamine, K_2_CO_3_, and DMF, 1 h.

One of the standard procedures for the preparation of amides was published by Xiao et al. in 2014 [67]. The carboxyl group was activated by 2-(1H-benzotriazole-1-yl)-1,1,3,3-tetramethyluronium tetrafluoroborate (TBTU). The reaction gave stable intermediate **12** in excellent yield and purity, and the follow-up reaction with propargyl amine under basic conditions afforded amide **13** in 1 h in 92% yield (Scheme 2). A similar experimental setup was used in 2020 by Li et al. [68] for the preparation of rhodamine B-based fluorescent probes for mechanistic study.

**Scheme 3:**
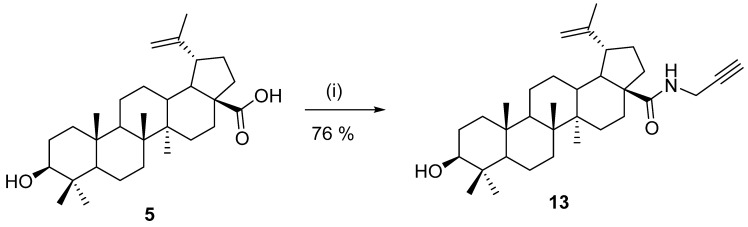
Preparation of a propargylamide derivative of betulinic acid [64]. Reagents and conditions: (i) propargylamine, DCC, HOBt, DIPEA, and DMF, 12 h.

Another procedure used by Dang Thi et al. [64] utilized the DCC/HOBt technique for the activation of carboxyl moieties. The exact conditions are described in Scheme 3.

Another protocol for amide preparation was used in 2015 by Wiemann et al. [69]. The authors prepared a series of hydroxamate derivatives of betulinic acid (**5**) derived containing amide and ester bonds. First, the hydroxyl group of **5** was protected in the form of an acetyl group. Next, oxalyl chloride was utilized for the activation of the carboxylic group. The in situ-generated acyl chloride was then treated with a hydroxamate derivative to provide appropriate amides. Amides were prepared in high yields of 68–90%.

Carbonyldiimidazole is another coupling reagent used frequently for the preparation of amide bonds. For example, in 2015, amide derivatives of betulinic acid (**5**) with a heterocyclic moiety were prepared by Cui et al. [70] in 73–83% yields.

**Figure 3:**
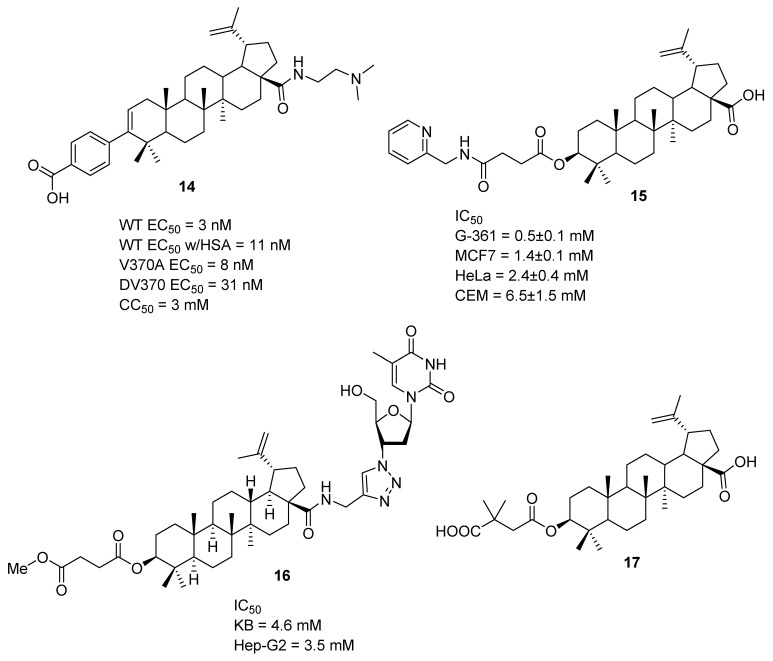
Selected derivatives of betulinic acid (**5**) with anti-HIV and anticancer activities.

Esters of betulinic acid (**5**) with dicarboxylic acids at their 3-hydroxy group (Figure 3) can also possess interesting biological activity, and esterification is one of the most common chemical transformations, for which enzymatic catalysis may be beneficial. A good example of an ester with important biological activity is the dimethylsuccinyl ester of betulinic acid, bevirimat (**17**), which was tested in Phase I and Phase II trials for its activity against HIV-1 infection [71]. Bevirimat was prepared using 2,2-dimethylsuccinic anhydride with DMAP in pyridine at a 70% yield by Hashimoto et al. in [72] 1997. The authors also prepared other ester derivatives using this procedure by exchanging 2,2-dimethylsuccinic anhydride with other anhydrides.

**Scheme 4:**
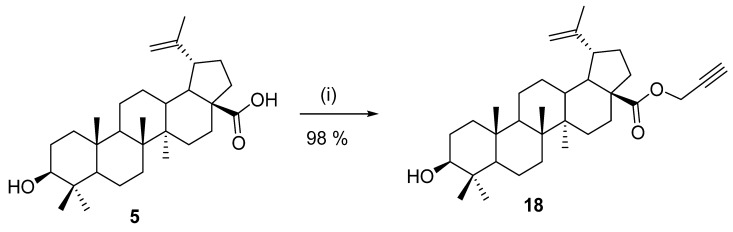
Preparation of a propargyl ester derivative of betulinic acid [73]. Reagents and conditions: (i) potassium carbonate, and acetone under reflux, 14 h.

Esters of betulinic acid (**5**) at their 28-COOH group can also be prepared by alkylation procedures with appropriate halogen derivatives in the presence of a base. For example, in 2016, Khan et al. [73,74] synthetized propargyl betulinate **18** using the conditions described in Scheme 4. Compound **18** was then used for the preparation of a large set of new triazole derivatives by 1,3-cycloaddition reactions.

Methyl betulinate can be prepared by alkylation with methyl iodide [75] or trimethylsilyldiazomethane [76] in good yield (higher than 80%). Another effective method for the preparation of methyl betulinate is the reaction of betulinic acid (**5**) with diazomethane in diethyl ether [74].

**Scheme 5:**
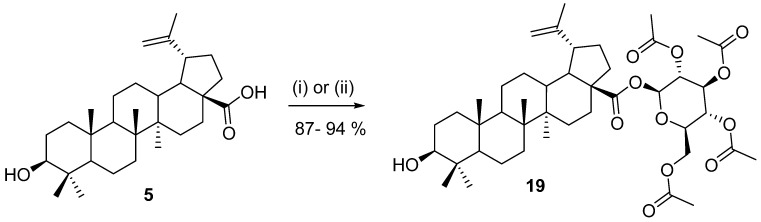
Preparation of glycoside of betulinic acid [81,82]. Reagents and conditions: (i) potassium carbonate, acetone, and acetonitrile, 20 °C, 96 h; (ii) silver(l) oxide, and pyridine, 3 h.

The preparation of triterpenic glycosides and sugar esters is another important chemistry method that may be performed using enzymes. Glycosides or sugar esters are usually more soluble in water than pure aglycones, and it was observed that in many cases, they retain biological activity and become more bioavailable [77]. This represents a significant improvement in pharmacological parameters of the potential drug candidate. The synthesis of both sugar esters and glycosides is usually complicated, and harsh methods are sometimes used, of which only some may be applied in the chemistry of triterpenes [78,79,80]. An example of the preparation of the glycoside **19** is shown in Scheme 5 [81,82]. According to our work, these methods have only limited use because they only work for certain sugars, while in other cases, the reactions fail or produce inseparable mixtures of isomers. Often, the reaction conditions need to be patiently optimized [77]. This preparation is a typical case in which chemoenzymatic synthesis would be very helpful.

## 6. Biocatalysis

According to the summary presented in an article by Milner and Maguire in 2012 [83], biocatalysis is a field that involves the participation of enzymes or whole cells that contain the desired enzyme or enzyme systems as catalysts for chemical reactions. In another article from 2017 by Sun et al. [84], the importance of biocatalysis for pharmaceutical synthesis was highlighted. The importance of biocatalysis lies mainly in the ability of biocatalysts to make the synthetic routes shorter. This can increase the overall yield of the reaction sequence. Enzymatically catalyzed reactions can often be executed under mild reaction conditions. Usage of toxic reagents can be avoided. Reactions catalyzed by biocatalysts can provide high yields with excellent chemo-, regio-, and stereoselectivity, and another important advantage is that undesired side products are often generated in smaller yields than with the use of classical organic synthesis. The main classes of biocatalysts are reductases, oxidases, hydrolases, lyases, isomerases and transaminases.

## 7. Biosynthesis of Pentacyclic Triterpenes

Biosynthesis of cyclic triterpenes starts from squalene (**20**) or squalene oxide (**21**) and is catalyzed by triterpene cyclases. Cyclisation is usually initiated by protonation of the terminal π bond of squalene or the terminal epoxide moiety of squalene oxide, and it continues with a cascade of carbon−carbon bond-forming reactions, ultimately yielding various triterpenes depending on the catalyst. The triterpene cyclase active site also must enforce appropriate conformation to the substrate to obtain correct chirality of the product. Squalene-hopene cyclase produces triterpene hopene from squalene, which is the starting material for other hopanoids. Oxidosqualene cyclase is responsible for the cyclization of squalene oxide [85]. The next section of this review is focused mainly on the biosynthesis of biologically interesting lupane-type triterpenoinds. Other compounds, such as oleanane and ursane pentacyclic triterpenoids, are not included because in 2020, Luchnikova et al. published a comprehensive review on their biosynthesis and biotransformation, distribution in nature, and biotechnological synthesis using microorganisms [18]. The review also contains information about selected biological activities of oleanane and ursane triterpenoids [18].

## 8. Biosynthesis of Betulinic Acid (5), Betulin (8), and Lupeol (9)

**Scheme 6:**
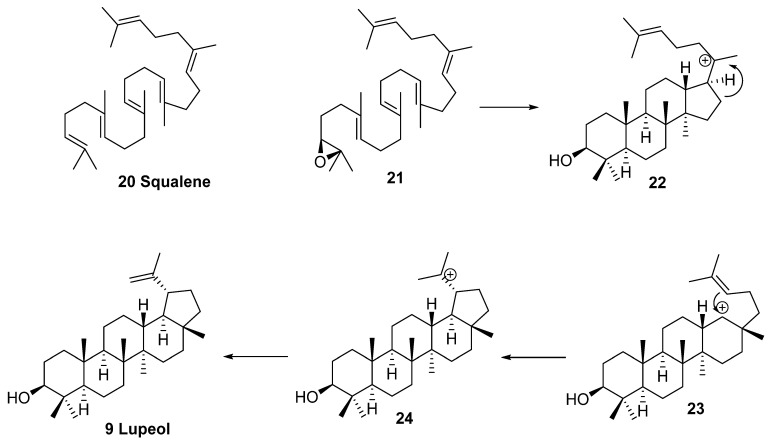
Mechanism of lupeol (**9**) biosynthesis [86].

Enzymatic synthesis of cyclic triterpenes, including lupeol (**9**), which is an intermediate for the biosynthesis of betulin (**8**) and betulinic acid (**5**), was reviewed in detail by Abe in 2007 [86]. The biosynthesis of lupeol (**9**) starts from (3*S*)-2,3-oxidosqualene (**21**). First, the cyclization of (3*S*)-2,3-oxidosqualene is initiated by protonization and produces the 6.6.6.5-fused tetracyclic dammarenyl C-20 cation **22**. Next, D-ring expansion occurs, producing cation **23,** from which lupanyl tertiary cation **24** is generated, and this charged species then eliminates protons from one of the terminal methyl groups to give lupeol (**9**) (Scheme 6) [86].

**Figure 4:**
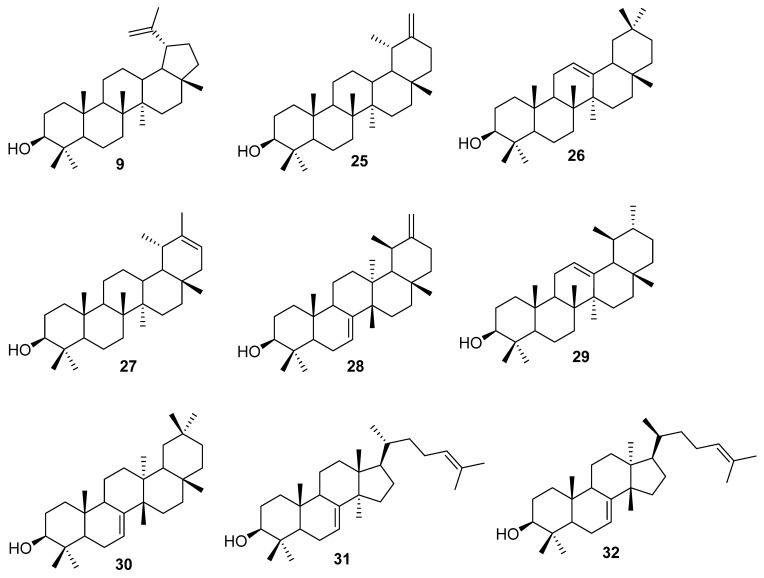
Chemical structures of lupeol (**9**), taraxasterol (**25**), β-amyrin (**26**), Ψ-taraxasterol (**27**), bauerenol (**28**), α-amyrin (**29**), multiflorenol (**30**), butyrospermol (**31**), and tirucalla-7,21-dien-3β-ol (**32**).

The next part of this section contains information about reported lupeol triterpene synthases. This topic was recently reviewed in detail in An et al. [87]; therefore, we will not discuss it in detail. In 1998, Herrera et al. [88] cloned and characterized the lupeol synthase gene from *Arabidopsis thaliana*. Expression of the LUP1 gene produced the major product lupeol (**9**) and minor amounts of β-amyrin (**26**) and other triterpene alcohols. In 1999, Shibuya et al. [89] found two new lupeol synthase cDNA genes using PCR. One gene was cloned from olive leaves of *Olea europaea* and coded OEW. The second gene, TRW, was cloned from dandelion roots of *Taraxacum officinale*. Expression of these genes in an ERG7-deficient yeast mutant leads to the accumulation of lupeol (**9**). This result confirmed that both genes encode lupeol synthase proteins. In 2000, Kushiro et al. [90] described the presence of multifunctional triterpene synthase in *Arabidopsis thaliana*. The presence of this enzyme was demonstrated by the expression of YUP8H12R.43 (from *Arabidopsis thaliana*) in yeast, leading to the production of at least nine terpenes. The authors were able to identify lupeol (**9**), taraxasterol (**25**), β-amyrin (**26**), Ψ-taraxasterol (**27**), bauerenol (**28**), α-amyrin (**29**), multiflorenol (**30**), butyrospermol (**31**), and tirucalla-7,21-dien-3β-ol (**32**) (Figure 4).

Another multifunctional triterpene synthase was described the same year by Morita et al. in *Pisum sativum*, which was discovered by expressing PSM. A mix of β-amyrin (**26**), α-amyrin (**27**), and several other minor triterpenes (lupeol (**9**) was one of them) was produced. In 2003, another four cDNA genes of oxidosqualene cyclase were cloned using the PCR method by Zhang et al. [91] from cell suspension cultures of *Betula platyphylla*. These genes were BPX, BPX2, BPW, and BPY. Expression was tested in *Saccharomyces cerevisiae*. Analyses showed that BPX and BPX2 are responsible for coding cycloartenol synthase, while BPW and BPY products are responsible for coding lupeol (**9**) and β-amyrin (**26**) synthases. An additional study focused on oxidosqualene cyclase from *Arabidopsis thaliana* was performed in 2003 by Ebizuka et al. [92]. Two new cDNAs, F1019.4 and T30F21.16, were identified by the authors. The authors were able to identify three biosynthetic products of expression of T30F21.16. These products included lupeol (**9**), bauerenol (**28**), and α-amyrin (**29**). Analytical data on the products of expression of F1019.4 showed the presence of tirucalla-7,21-diene-3β-ol (**32**). In 2003, Iturbe-Ormaetxe et al. [93] focused on cloning and characterization of three triterpene synthases from *Medicago truncatula* and *Lotus japonicus*. Expression of LjAMY2 in yeast produced β-amyrin (**26**) and lupeol (**9**) in almost equal amounts according to results presented by the authors. In 2004, Hayashi et al. [94] attempted to elucidate the regulation of the production of triterpenoids in *Glycyrrhiza glabra*. The authors were able to deduce lupeol synthase and oxidosqualene cyclase cDNA responsible for the accumulation of lupeol (**9**) in pYES2-GgLUS1. The cDNA was termed GgLUS1. In 2006, Guhling et al. [95] also investigated the biosynthesis of triterpenoinds in the stems of *Ricinus communis*. Cloning of two oxidosqualene cyclases and their expression in yeast led to characterization of a cycloartenol synthase (RcCAS) and a lupeol synthase (RcLUS). The expression of RcLUS matched the accumulation of cuticular lupeol (**9**) in castor beans. Another analysis of the functions and structures of oxidosqualene cyclase genes of the model *Lotus japonicus* was performed by Sawai et al. in 2006 [96]. Two genes were recognized for the production of lupeol (**9**). The OSC3 gene is responsible for the production of lupeol (**9**), and OSC8 is responsible for the production of lupeol (**9**) and β-amyrin (**26**). In 2006, Basyuni et al. [97] cloned triterpene synthase (KcMS) from *Kandelia candel*. Expression in yeast produced a mixture of lupeol (**9**), β-amyrin (**26**), and α-amyrin (**29**) in a 2:1:1 ratio. In 2007, Basyuni et al. [98] contributed to the identification of genes responsible for the production of lupeol (**9**). Gene expression of BgLUS and BgbAS from *Bruguiera gymnorrhiza* resulted in the production of lupeol (**9**) and β-amyrin (**26**). Expression of RsM1 from *Rhizophora stylosa* produced germanicol, β-amyrin (**26**), and lupeol (**9**) at a ratio of 63:33:4, and expression of RsM2 from *Rhizophora stylosa* produced taraxerol, β-amyrin (**26**), and lupeol (**9**) at a ratio of 70:17:13. In 2010, Wang et al. [99] described that the expression of KdLUS from *Kalanchoe daigremontiana* produced lupeol (**9**). In 2012, Yin et al. [100] published a study dealing with the distribution of betulin (**8**) and oleanolic acid (**7**) in various organs of white birch (*Betula platyphylla*) at different ages. As part of their study, the authors determined the expression of 4 OSC genes (LUS, β-AS, CAS1, and CAS2) involved in the triterpenoid synthesis pathways by real-time RT-PCR. In 2015, in a study focused on *Barbarea vulgaris*, Khakimov et al. [101] identified two 2,3-oxidosqualene cyclases that produce triterpenes. The main product of LUP2 is lupeol (**9**), and LUP5 produces β-amyrin (**26**) and α-amyrin (**29**).

To obtain betulin (**8**) and betulinic acid (**5**) from lupeol (**9**) in the next step, oxidation of the methyl group at C-28 must be performed. The enzymes responsible for this oxidation belong to the class of cytochrome P450 enzymes. This topic was also reviewed in detail in An et al. [87]. In 2011, Fukushima et al. [102] presented a study focused on gene analysis of *Medicago truncatula*. The authors found a correlation between CYP716A12 and β-amyrin synthase. The in vitro assay performed by these researchers confirmed that CYP716A12 can oxidize β-amyrin at position C-28 to produce oleanolic acid (**7**). Another confirmation was performed using in vivo testing in transgenic yeast that can produce β-amyrin by expressing CYP716A12. According to the authors, CYP716A12 was also able to produce betulinic acid (**5**) by oxidation of lupeol (**9**) via betulin (**8**), and ursolic acid (**7**) by oxidation of α-amyrin. In addition, the authors identified homologs of CYP716A12 in grapes encoding CYP716A15 and CYP716A17 that can also participate in the biosynthesis of triterpenes. In 2012, Huang et al. [103] isolated two cDNAs from *Catharanthus roseus.* The CrAS gene is responsible for encoding 2,3-oxidosqualene cyclase, and the CrAO gene is responsible for encoding amyrin C-28 oxidase. Analysis in *Saccharomyces cerevisiae* CrAO confirmed that this oxidase is able to convert α-amyrin (**29**), β-amyrin (**26**) and lupeol (**9**) to ursolic acid (**6**) and oleanolic acid (**7**) by coexpressing CrAS and CrAO. Expressing AtLUP1 from *Arabidopsis thaliana* instead of CrAS and CrAO in yeast produced betulin (**8**) and betulinic acid (**5**) and small amounts of oleanolic acid (**7**). In 2015, Khakimov et al. [101] also described cytochrome P450 (CYP72As) in addition to the previously mentioned 2,3-oxidosqualene cyclases in their work focused on *Barbarea vulgaris*, which produces triterpenes. Two cytochrome P450s (CYP716A80 and CYP716A8) were expressed in *Saccharomyces cerevisiae*. These cytochrome P450s were coexpressed with *Lotus japonicus* β-amyrin synthase to provide β-amyrin as a substrate. The main products were oleanolic acid (**7**), erythrodiol, and two unknown oxygenated compounds of β-amyrin. Expression of selected *Barbarea vulgaris* OSCs, P450s, and UGTs in *Nicotiana benthamiana* produced varying levels of oleanolic acid (**7**), ursolic acid (**6**), and betulinic acid (**5**), which are derived through C-28 oxidation of β-amyrin, α-amyrin, and lupeol (**9**). In 2016, Zhou et al. [104] reported a gene encoding BPLO that was responsible for encoding a lupeol C-28 oxidase from *Betula platyphylla*. The authors showed high activity of this gene in betulinic acid (**5**) biosynthesis. The use of this oxidase will be further described in a section focusing on the biocatalyzed production of betulinic acid (**5**). In 2017, Tamura el al. [105] reported that CYP716A179 of *Glycyrrhiza uralensis* functions as a triterpene C-28 oxidase. Coexpression of various gene combinations in yeast produced triterpenoids depending on the starting material. Coexpression of LUS, CPR, and CYP716A179 in yeast betulin (**8**), betulinic aldehyde (**11**), and betulinic acid (**5**) was produced from lupeol (**9**). In 2019, Huand et al. [106] reported that RoCYP01 (CYP716A155) from *Rosmarinus officinalis* is able to oxidase to convert lupeol (**9**) into betulinic acid (**5**). The use of this oxidase will be further described in a section focusing on the biocatalyzed production of betulinic acid (**5**). Suzuki et al. [107] reported one oxidosqualene cyclase and two cytochrome P450 enzymes using expression in yeast. Coexpression of LUS/CPR/CYP716A51 produced betulinic acid (**5**). Betulin (**8**) was also detected. Other combinations may lead to different products.

## 9. Biocatalyzed Production of Lupane Triterpenoids—Betulinic Acid (5), Betulin (8), and Lupeol (9)

As mentioned earlier, whole cells can be used for the preparation of valuable compounds. This section contains information about progress made in the production of betulinic acid (**5**) using microorganisms, which was also partly reviewed in An et al. [87]. Here we include several new articles and articles focused on betulin (**8**) and lupeol (**9**). This section is summarized in Table 2. In 2011, Liu et al. [108] reported optimization of the biotransformation of betulin (**8**) to betulinic acid (**5**) catalyzed by the fungus *Armillaria luteovirens* Sacc ZJUQH100-6. Tween 80 and the substrate concentration were identified as significant factors. The optimum conditions were observed at pH 6.0 with 0.57% Tween 80, 15 mg l(-1) betulin (**8**), and 3 d of inoculation. The highest productivity of betulinic acid (**5**) predicted according to the authors was 9.32%, which was increased by 74.53% compared with that of the nonoptimized compound. The authors also experimentally compared the bioconversion of betulin (**8**) and betulin-28-monooxygenase activities between the optimized and the nonoptimized conditions. In 2012, Bai et al. [109] described an optimization study for betulin (**8**) production from *Inonotus obliquus*. The most significant variables of the medium components were glucose, yeast extract, and MgSO_4_. The optimal temperature was 25 °C, and the optimal initial pH was 6.0. The optimal concentrations for betulin (**8**) production were 30 g/L glucose, 3.5 g/L yeast extract, and 5 mmol/L MgSO_4_·7H_2_O. Under optimal conditions, the betulin (**8**) concentration in a 5 L stirred-tank bioreactor reached 69.37 mg/L. The authors also indicated that mycelial growth and pellet morphology may be critical parameters for betulin (**8**) production. In 2014, Wang et al. [110] proved that increased production of betulin (**8**) and other natural compounds in *Inonotus obliquus* can be induced in the presence of aqueous extract and methanol extract from birch bark. *Saccharomyces cerevisiae* is one of the most important species of yeast and is widely used in the production of ethanol (alcoholic beverages) and bakery products [111]. In an article by Li et al. [112], the possibility of the production of betulinic acid (**5**) in *Saccharomyces cerevisiae* was investigated. The authors inserted genes for the synthesis of betulinic acid (**5**) into yeast cells and regulated their expression to find an optimum ratio of carbon flux between the metabolic pathway leading to betulinic acid (**5**) and the natural pathway leading to fatty acids (lipids). The optimum expression levels of genes in both pathways yielded a stable yeast culture efficiently producing betulinic acid (**5**). The yields of **5** varied within the range from 0.01 to 1.92 mg L^−1^ OD^−1^. Another study focused on increasing the yield of **5** produced by *Saccharomyces cerevisiae* was published in 2015 [113]. The authors hypothesized that intracellular supply of NADPH/oxygen could improve the yield of betulinic acid (**5**). To test this hypothesis, the expression of mutated 2,3-butanediol dehydrogenase (mBDH1) and yeast codon-optimized Vitreoscilla hemoglobin (mvhb) was evaluated. The results showed that the final concentrations of betulinic acid (**5**) were 1.5 and 3.2 times higher. The expression of mvhb was also responsible for the inhibition of yeast growth. An appropriate concentration of acetoin with the expression of mBDH1 was able to maintain desirable yeast growth. Next, improvement in the production of betulinic acid (**5**) in *Saccharomyces cerevisiae* was reported in 2016 by Zhou et al. [104]. The authors were able to identify lupeol C-28 oxidase from *Betula platyphylla*, as mentioned in the previous section. This oxidase showed high activity in the biosynthesis of betulinic acid (**5**). Attention was also paid to the comparison of two yeast strains producing **5**. The WAT11 strain was evaluated as the most effective option because of its better conversion of betulin (**8**) to betulinic acid (**5**) compared to the CEN.PK strain. The authors were also able to construct a Gal80p mutant that produced 0.16 mg/L/OD_600_ betulinic acid (**5**). For comparison, the wild strain produced only 0.07 mg/L/OD_600_.

In 2016, Lin et al. [114] obtained cell factories for the production of lupeol (**9**) in *Saccharomyces cerevisiae* by increasing the supply of squalene using the DNA assembler method and by integrating *Arabidopsis thaliana* lupeol (**9**) synthesis genes into the chromosomes of strains. The authors reported that the cell factories could produce 8.23 mg/L of lupeol (**9**). In 2017, Czarnotta et al. [115] reported fermentation and purification methods for the preparation of betulinic acid. *Saccharomyces cerevisiae* CEN.PK BA4 was used for the process. Excess ethanol was key for fermentation in nitrogen-limited resting cells. Purification was performed using solid-liquid extraction without prior cell disruption. The yield of betulinic acid (**5**) was 182 mg/L. According to the authors, further metabolic engineering of the host is required because of low specific productivity and product specificity. In 2017, Arendt et al. [116] reported the production of betulinic acid (**5**) and its intermediates lupeol (**9**), betulin (**8**), and betulinic aldehyde (**10**). According to the authors, the production of betulinic acid (**5**) and 3β,20-dihydroxylupane was significantly increased in the pah1 yeast strain after coexpressing lupeol (**9**) synthase from *A. thaliana* (AtLUP1) with the C28-oxidase CYP716A83 from *C. asiatica*. In 2019, D’Adamo et al. [117] published pioneering work in the engineering of the unicellular alga *Phaeodactylum tricornutum*. Introducing *Lotus japonicus* oxidosqualene cyclase and *Medicago truncatula* cytochrome P450 along with its native reductase enabled the production of betulin (**8**) and its precursor lupeol (**9**). In 2019, Qiao et al. [118] presented biosynthetic production of lupeol (**9**) in *Escherichia coli* and *Saccharomyces cerevisiae* cells by recruiting three optimized lupeol pathway genes from different organisms. The authors introduced squalene synthase from *Thermosynechococcus elongates*, squalene epoxidase from *Rattus norvegicus* and lupeol synthase from *Olea europaea* into *E. coli* BL21(DE3). The evaluation showed high activities. Next, the reconstituted lupeol pathway was transferred into two different yeast strains, WAT11 and EPY300, and they were both compared. EPY300 showed 4.6–9.4-fold higher lupeol (**9**) production than WAT11. The authors also developed a highly lupeol-producing yeast strain, named ECHHOe. The maximum lupeol (**9**) titer after 72 h of flask cultivation reached 200.1 mg/L, which was 24.4-fold higher than that of a previously reported strain. *Saccharomyces cerevisiae* is not the only yeast investigated for the production of betulinic acid (**5**). *Yarrowia lipolytica* is one of the most studied yeast species and is capable of synthesizing valuable metabolites according to information from an article published in 2019 [119]. In 2019, Sun et al. [120] reported the biosynthesis of betulinic acid (**5**) in *Yarrowia lipolytica*. Substitution of glucose with glycerol as a starting material leads to an increase in betulinic acid (**5**) production. A yield of 26.53 mg/L acid **5** was achieved with 40 g/L glycerol. The use of glycerol led to an increase in the expression of key genes in biosynthesis and increased the supply of acetyl-CoA. Another increase in the production of betulinic acid (**5**) by *Yarrowia lipolytica* was published by Jin et al. [121]. The systematic engineering undertaken by these researchers led to a yield of 204.89 ± 11.56 mg/L triterpenoids. The percentage of betulinic acid (**5**) in this yield was 23.71%. In the same year, Hung et al. [106] identified oxidase RoCYP01 (CYP716A155) in *Rosmarinus officinalis*. This oxidase was able to effectively oxidase lupeol (**9**) to betulinic acid (**5**). The authors were able to construct a yeast strain that provided yields of betulinic acid higher than 1 g/L. In 2020, Gowers et al. [122] used the SCRaMbLE technique (system of inducible in vivo deletion and rearrangement of synthetic yeast chromosomes) to optimize yeast strains to produce betulinic acid (**5**). Automated sample preparation, an ultrafast LC-MS method, and barcoded nanopore sequencing were combined to rapidly isolate and characterize the best performing strains. The semiautomated workflow used by the authors screened 1000 colonies. These researchers identified and sequenced 12 strains with improvements from 2- to 7-fold in the betulinic acid titer.

**Table 2:** Biocatalyzed production of lupane triterpenoids—overview.

Microorganisms	Year	Modification of Biosynthesis	Production	Reference
*Armillaria luteovirens* Sacc	2011	optimization of various conditions	production of **5**	[108]
*Inonotus obliquus*	2012	optimization of various conditions	69.37 mg/L of **8**	[109]
*Inonotus obliquus*	2014	yield increase with aqueous extract and methanol extract from birch bark	increased production of **8**	[110]
*Saccharomyces cerevisiae*	2014	insertion and expression regulation of genes	**5** from 0.01 to 1.92 mg L^−1^ OD^−1^	[112]
*Saccharomyces cerevisiae*	2015	changes in intracellular supply of NADPH/oxygen	1.5 and 3.2 times higher production of **5**	[113]
Gal80p mutant of *Saccharomyces cerevisiae*	2016	up-regulation of the expressed genes	0.16 mg/L/OD_600_ of **5**	[104]
*Saccharomyces cerevisiae*	2016	increase in the supply of squalene	8.23 mg/L of **9**	[114]
*Saccharomyces cerevisiae* CEN.PK BA4	2017	usage of excess of ethanol	182 mg/L of **5**	[115]
	2017			[116]
*Phaeodactylum tricornutum*	2019	introduction of *Lotus japonicus* oxidosqualene cyclase and *Medicago truncatula* cytochrome P450 with native reductase	production of **8** and **9**	[117]
*Escherichia coli* and *Saccharomyces cerevisiae*	2019	usage of optimized lupeol pathway genes	production of **9**	[118]
*Yarrowia lipolytica*	2019	usage of glycerol as a starting material	26.53 mg/L of **5**	[120]
*Yarrowia lipolytica*	2019	systematic metabolic engineering	204.89±11.56 mg/L of triterpenoids (23.71% of **5**)	[121]
*Saccharomyces cerevisiae*	2019	usage of RoCYP01 (CYP716A155)	yields of **5** higher than 1 g/L	[106]
various yeast strains	2020	SCRaMbLE technique	production of **5**	[122]

## 10. Enzymatic Modification of Betulinic Acid (5) and Betulin (8)

**Scheme 7:**
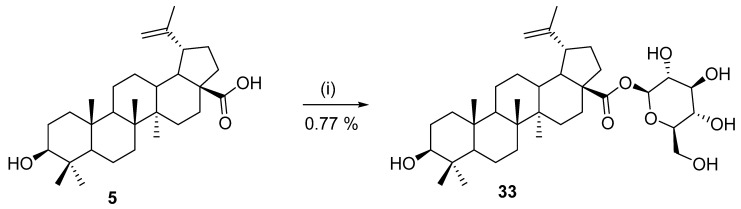
Preparation of 28-*O*-β-D-glucopyranosyl 3β-hydroxy-lup-20(29)-en-28-oate (**25**) [123]. Reagents and conditions: (i) β-D-glucose, resting-cell suspensions of *Cunninghamella species*, N,N-dimethyl-formamide, and phosphate buffer (pH 7.2), 216 h.

Selected chemical methodologies for amidation, esterification, and hydrolysis have already been mentioned in the previous section. This section contains information about chemoenzymatic modification of derivatives of betulinic acid (**5**), betulin (**8**), and lupeol (**9**). In 1999, Chatterjee et al. [123] investigated the metabolic biotransformation of betulinic acid (**5**) in a selected fungal model system (*Cunninghamella* species NRRL 5695). This investigation led to the discovery of the conjugate 28-*O*-β-d-glucopyranosyl 3β-hydroxy-lup-20(29)-en-28-oate (**33**) (Scheme 7). Thirteen other fungal cultures were also tested by the authors for the preparation of the same compound, but only *Cunninghamella* species NRRL 5695 exhibited the ability to produce the exact glucopyranosyl ester **33**. Biological evaluation of its cytotoxic activity against several human melanoma cell lines showed no interesting activity in comparison with betulinic acid (**5**) [123].

**Scheme 8:**
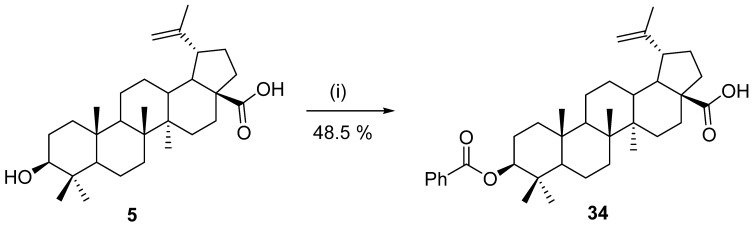
Optimal conditions for enzymatic esterification of betulinic acid (**5**) with benzoyl chloride [124]. Reagents and conditions: (i) PhCOCl, Novoenzym 435 (123 mg), and chloroform, 51.5 °C, 10.7 h.

In 2008, Yasin et al. published work in which response surface methodology was used to determine the optimal conditions for enzymatic synthesis of betulinic acid benzoyl ester **34**. Novoenzym 435, benzoyl chloride, and betulinic acid (**5**) were used as model components for their study. The experimental yield was 48.5% under the optimal conditions (Scheme 8) [124].

**Scheme 9:**
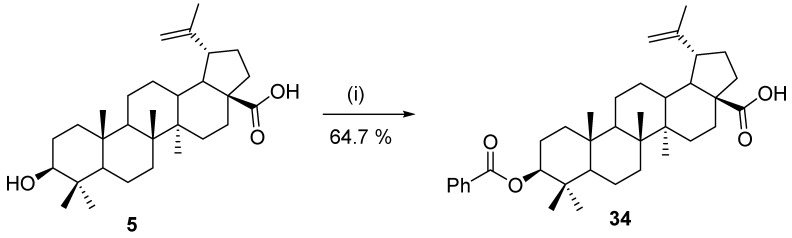
Optimal conditions for enzymatic esterification of betulinic acid (**5**) with acid anhydride [125]. Reagents and conditions: (i) phthalic anhydride (1.11 eq.), K_2_CO_3_, celite 545, Novoenzym 435 (145.6 mg), and chloroform:hexane/(1:1), 53.9 °C, 20.3 h.

Using the same methodology, two years after the first pioneering work with Novoenzym 435, Ahmad et al. published another optimization study of the synthesis of ester **34**. Instead of acyl chloride, phthalic anhydride was used as the acylating reagent, and CHCl_3_/hexane was used as the solvent system. The experimental yield was 64.7% under the optimal conditions (Scheme 9) [125].

**Scheme 10:**
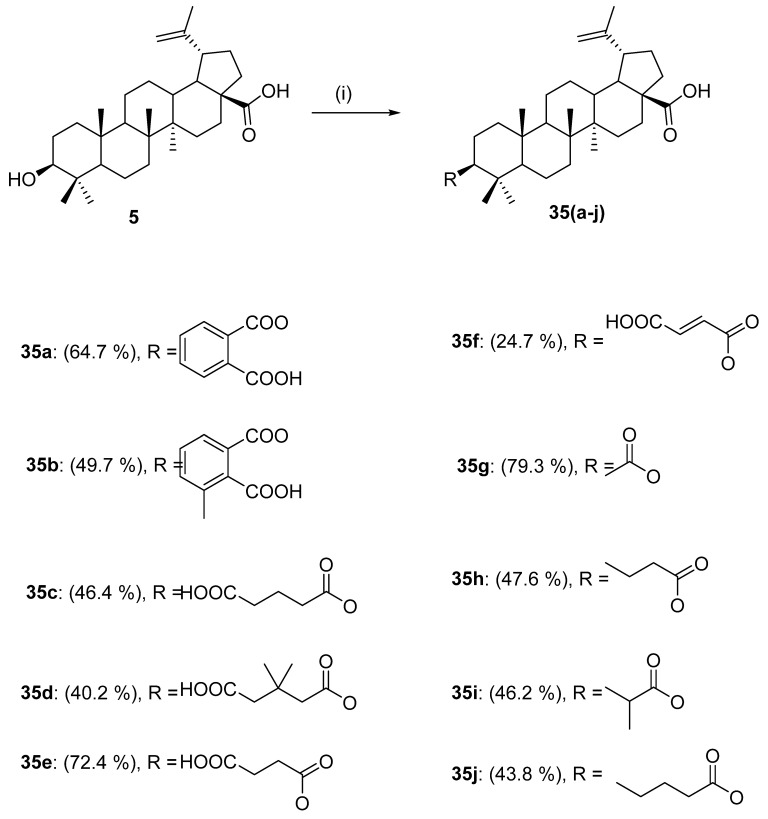
Synthesis of ester derivatives of betulinic acid by enzyme catalysis [126]. Reagents and conditions: (i) acid anhydride, celite 545, K_2_CO_3_, chloroform:hexane/(1:1), and Novoenzym 435, 54 °C, 20 h.

In the same year, Ahmad et al. also published an article dealing with the preparation of 3-*O*-acylated betulinic acid derivatives **35a**–**35j** catalyzed by Novoenzym 435. Appropriate anhydrides were used as acylating reagents (Scheme 10). The anticancer activity of these derivatives was also evaluated in this study in vitro against human lung carcinoma (A549) and human ovarian (CAOV3) cancer cell lines. The most promising results were shown by 3-*O*-glutarylbetulinic acid **35j**, 3-*O*-acetyl-betulinic acid **35g**, and 3-*O*-succinyl-betulinic acid **35e** against the A549 cancer cell line (IC_50_ < 10 μg/mL). Both derivatives had better cytotoxic activity than betulinic acid (**5**) [126].

**Scheme 11:**
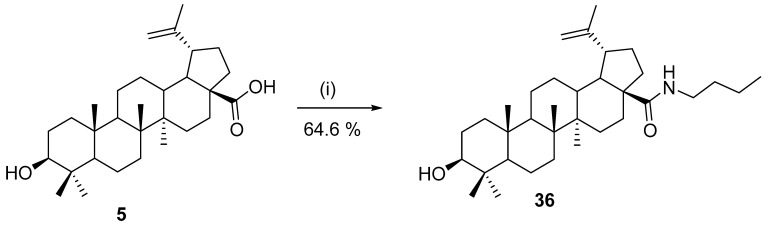
Optimal conditions for enzymatic amidation of betulinic acid [128]. Reagents and conditions: (i) butyl amine (1 eq.), Novoenzym 435 (100 mg), and chloroform:hexane/(9:1), 40 °C, 24 h.

In 2012, Mao et al. [127] reported the biotransformation of betulin (**8**) by the yeast strain *Rhodotorula mucilaginosa*. This yeast converted 52.65% of the added 4 mg/mL betulin to betulone and 11,14-octadecadienoic acid methyl ester under optimal conditions (initial pH 6.0, 20 °C for 1 d). In 2016, Yusof et al. optimized the conditions for the enzymatic synthesis of betulinic acid amide **36**. Novoenzym 435 from *Candida antartica* was used as a catalyst for enzymatic amidation, and butylamine was used as an amine reagent that was coupled with betulinic acid (**5**). Using the optimal conditions gave the product in 64.6% yield (Scheme 11) [128].

In 2017, Guo et al. reported a one-step enzymatic synthesis of liposomes of folate–poly(ethyleneglycol)_3400_–cholesterol conjugates. The prepared liposomes were loaded with betulinic acid (**5**) by the thin lipid film method. In vitro testing on HepG2 cells showed enhanced cytotoxic activity of folate-functionalized liposomes (IC_50_ = 63.07 ± 2.22 μg/mL) compared to normal liposomes loaded with betulinic acid (**5**) (IC_50_ = 93.14 ± 2.19 μg/mL) [129]. In 2019, Dai et al. [130] identified a novel CYP enzyme that catalyzes C-2α hydroxylation in *Crataegus pinnatifida*. It can oxidize oleanane-, ursane- and lupane-type pentacyclic triterpenoids. Application in yeast led to the production of 384, 141, and 23 mg/L maslinic acid (a derivative of oleanolic acid [7]), corosolic acid (a derivative of oleanolic acid [7]), and alphitolic acid (a derivative of betulinic acid), respectively.

## 11. Hydrolysis of Sugar Esters of Betulinic Acid

**Scheme 12:**
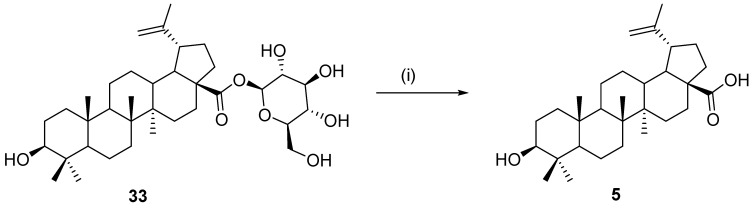
Enzymatic hydrolysis catalyzed by β-glucosidase [123]. Reagents and conditions: (i) ß-glucosidase, N,N-dimethyl-formamide, and phosphate buffer (pH 5), 37 °C, 72 h.

In 1999, Chatterjee and coworkers described enzymatic hydrolysis of a prepared derivative of betulinic acid **33** by the action of the enzyme β-glucosidase. Betulinic acid (**5**) was identified as a single product of this transformation [123] (Scheme 12).

**Scheme 13:**
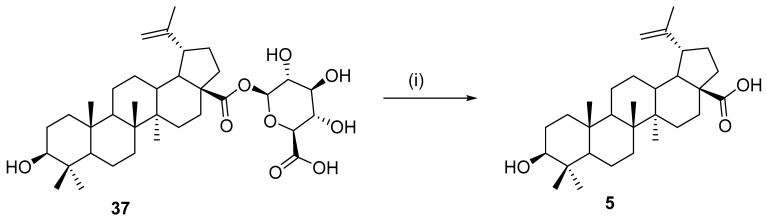
Hydrolysis to betulinic acid by the enzyme β-D-glucuronidase (*Escherichia coli*) [131]. Reagents and conditions: (i) *Escherichia coli* ß-D-glucuronidase, 37 °C, 1 h.

In 2009, Gauthier et al. described the preparation of 28-*O*-β-d-glucuronide betulinic acid **37** from peracetylated methyl glucuronate bromide under phase-transfer conditions. It was also described that derivative **37** could be used as a prodrug in anticancer treatment because of its noncytotoxicity, nonhemolyticity, and better water solubility, and it showed good in vitro stability in phosphate buffer. In vitro complete hydrolysis to betulinic acid could be achieved by the action of enzymatic β-d-glucuronidase (*Escherichia coli*) (Scheme 13) [131].

## 12. Microbial Oxidation of Betulinic Acid (5)

**Figure 5:**
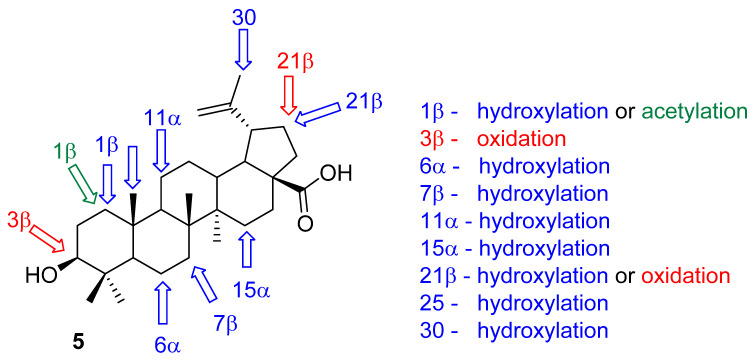
Positions of the Betulinic acid (**5**) which are most commonly modified by microbial oxidations using various fungal and bacterial species.

As previously mentioned, one of the most significant disadvantage of betulinic acid (**5**) is the low solubility and bioavailability. Most of the time, this problem is solved by the introduction of a polar moiety that is capable of improving of the pharmacological profile. Microbial transformations, however, offer another option for increasing the polarity, through introduction of hydroxyls in certain position of the triterpenic skeleton by enzymatic oxidation. This oxidation is usually performed by cytochromes P-450 in living bacteria or fungi. A great advantage of this procedure is that all microorganisms introduce the hydroxyls highly stereospecifically, and they are also very selective in which positions they modify. As early as in 2000, Chtterjee et al. published the use of *Bacillus megaterium* ATCC 13368 for the introduction of hydroxyls into the skeleton of betulinic acid (**5**) in positions 11α, 1β, 7β, and 15α [132]. The same research group in Kouzi et al. describe a more comprehensive approach, using three microorganisms—*Bacillus megaterium* ATCC 14581, *Cunninghamella elegans* ATCC 9244, and *Mucor mucedo* UI-4605—to introduce hydroxyls into the positions 1β, 6α, and 1β [133]. In 2007, Bastos et al. studied the metabolization of acid **5** in three fungi species (*Arthrobotrys*, *Chaetophoma,* and *Dematium*) and obtained products monohydroxylated in the position 7β, 15α, 25, or 30 [134]. Various strains of bacteria *Bacillus megaterium*, *Streptomyces fragilis*, *Cunninghamella elegans*, and *Aspergillus terreus* were used by Goswami et al. in 2015 to obtain a number of hydroxyanalogues of betulinic acid (**5**) in [135]. In 2021, two fungi species *Circinella muscae* and *Cunninghamella echinulate* were used to produce a number of hydroxyderivatives of acid **5** [136]. A summary of the possible transformations is shown in Figure 5.

## 13. Conclusions and Future Perspectives

Pentacyclic triterpenes are important natural products with a variety of biological activities that predetermine them as potential drugs. To optimize the properties (e.g., activity, solubility, bioavailability, and susceptibility to metabolism) of the original active molecules, structures usually need to be slightly modified. Most of the time, this modification has been achieved by standard methods of organic synthesis. At present, these methods enable almost unlimited varieties of chemical transformations, but they have several drawbacks. In some cases, harsh reaction conditions, such as high temperatures, long reaction times, and environmentally dangerous or toxic reagents, must be used. In other cases, it is almost impossible to obtain enantiomerically or diastereomerically pure compounds. In these cases, enzymatic synthesis or biocatalysis may offer a successful alternative. Enzymes usually work under mild conditions and produce pure enantiomers or diastereoisomers. This property is especially of interest in the synthesis of triterpenic saponines and prodrugs. This selectivity is also demonstrated in Section 12, where various species use different cytochromes P450 to introduce hydroxyls into the lupane skeleton with high regioselectivity and stereospecifity. A number of enzymes were developed to be able to perform the reactions in organic solvents, rather than only in water-based media. To date, enzymes have rarely been used to modify lupane triterpenoids, but they are often used in the synthesis of other analogs of pentacyclic triterpenes [18], and there is no reason why they should not work with lupanes. Along with the currently known and used esterification or amidation of lupane acids and their hydrolysis, there is much room for other reactions, such as redox reactions catalyzed by various cytochromes P450 to get specifically modified triterpenes in the ways described in Section 12 of this review. In addition, one may expect that there is great potential to mutate squalene cyclase to obtain completely new types of triterpenoid skeletons that are still unknown. These completely new, artificial triterpenoids may represent a large platform for entirely new terpenoid chemistry. For example, in 2013, Okamoto and Sato [137] described the formation of two unnatural pentacyclic triterpenes formed by head-to-tail cyclization from acyclic triterpene β-hexaprene using tetraprenyl-β-curcumene cyclase from *Bacillus subtilis*. In conclusion, enzymatic catalysis and biosynthesis in the field of lupane triterpenoids have not been fully elucidated and offer many interesting topics to study.

## Figures and Tables

**Table 1:** Main natural sources of lupane triterpenoids.

Plant Source	Year	Extraction Medium	Compounds	Reference
*Gratiola officinalis*	1902	methanol	**5**	[32]
*Vauquelinia corymbosa*	1976	chloroform	**5**	[33]
*Syzigium claviflorum*	1994	methanol	**5** and platanic acid	[34]
*Eucalyptus globulus*	2002	dichloromethane	**5** and other terpenoids	[37]
*Rosmarinus officinalis*	2002	methanol	**5**	[38]
*Betula platyphylla*	2006	various organic solvents	**5**; **8**	[42]
*Betula—various species*	2014	ethanol	**8**	[48]
*Platanus occidentalis*	2011	water and organic solvents	**8**	[43]
*Platanus hispanica*	2004	methanol	**5**	[44]
*Souroubea sympetala*	2011	carbon dioxide	**5**	[45]
*Eucalyptus grandis x globulus*	2013	supercritical carbon dioxide	**5** and other terpenic acids	[46]
purchased birch bark	2015	subcritical water	**5**	[41]
*Eucalyptus globulus*	2020	hydrophobic deep eutectic solvents	**5** and other terpenic acids	[47]

## Data Availability

Not applicable.
